# Current Advances in Technologies for Single Extracellular Vesicle Analysis and Its Clinical Applications in Cancer Diagnosis

**DOI:** 10.3390/bios13010129

**Published:** 2023-01-12

**Authors:** Lei Qiu, Xingzhu Liu, Libo Zhu, Liqiang Luo, Na Sun, Renjun Pei

**Affiliations:** 1 Department of Chemistry, College of Sciences, Shanghai University, Shanghai 200444, China; 2 Key Laboratory for Nano-Bio Interface, Division of Nanobiomedicine, Suzhou Institute of Nano-Tech and Nano-Bionics, Chinese Academy of Sciences, Suzhou 215123, China; 3 School of Nano-Tech and Nano-Bionics, University of Science and Technology of China, Hefei 230026, China

**Keywords:** extracellular vesicles, heterogeneity, single particle, cancer, diagnostic applications

## Abstract

Extracellular vesicles (EVs) have been regarded as one of the most potential diagnostic biomarkers for different cancers, due to their unique physiological and pathological functions. However, it is still challenging to precisely analyze the contents and sources of EVs, due to their heterogeneity. Herein, we summarize the advances in technologies for a single EV analysis, which may provide new strategies to study the heterogeneity of EVs, as well as their cargo, more specifically. Furthermore, the applications of a single EV analysis on cancer early diagnosis are also discussed.

## 1. Introduction

Extracellular vesicles (EVs) are actively produced by most types of cells, which have been categorized into three subgroups, based on their biological origin [1,2], release pathway [3] and size [4], including apoptotic vesicles (50–5000 nm) [5], microvesicles (100–1000 nm) [6,7] and exosomes (30–100 nm). EVs can be isolated from various biological fluids with minimal invasion, such as salivary [8], plasma [9], urine [10], breast milk [11] and seminal plasma [12], which has sparked considerable interest in cancer research. There are various cargos in EVs, including proteins [13], enzymes [14], DNA [15], mRNA [16], non-coding ribonucleic acid (ncRNA) and lipids, cytokines and growth factor receptors [17,18,19,20]. Importantly, tumor cell-derived EVs can induce the deterioration of non-malignant tissues [21] by modulating the tumor’s microenvironment, making it a potential circulating biomarker for cancer diagnosis [22,23,24], staging [25], treatment monitoring [26] and prognosis [27].

In the past decade, various methods for EV detection have been developed, but many of them focused on EV quantification or characterization in bulk [28]. For example, small-angle X-ray scattering (SAXS) has been used to reveal the details of the EV’s phospholipid bilayer structures and encapsulated transmembrane proteins [29,30]. However, it is limited to bulk analysis methods to reveal the high heterogeneity of the exosomes. As EVs are a group of heterogeneous particles, they have been considered to play an important role in tumor development. Moreover, there is also a huge challenge for the clinical application of EV detection in cancer diagnosis with a high degree of sensitivity, especially in early stages when only a small amount of EVs can be found in the liquid biopsy samples [31]. Recent studies suggest that single EV analysis techniques may provide a powerful tool to explore the diversity of EVs and address these challenges [32,33,34]. This review article summarizes the recent advances in the single EV analysis technology from the perspective of different analysis strategies, followed by a discussion on the applications of the single EV analysis technology in the field of cancer diagnosis.

## 2. Current Advances in the Single EV Analysis Techniques

### 2.1. Electron Microscopy-Based Methods for the Single EV Morphology Characterization 

**Figure 1:**
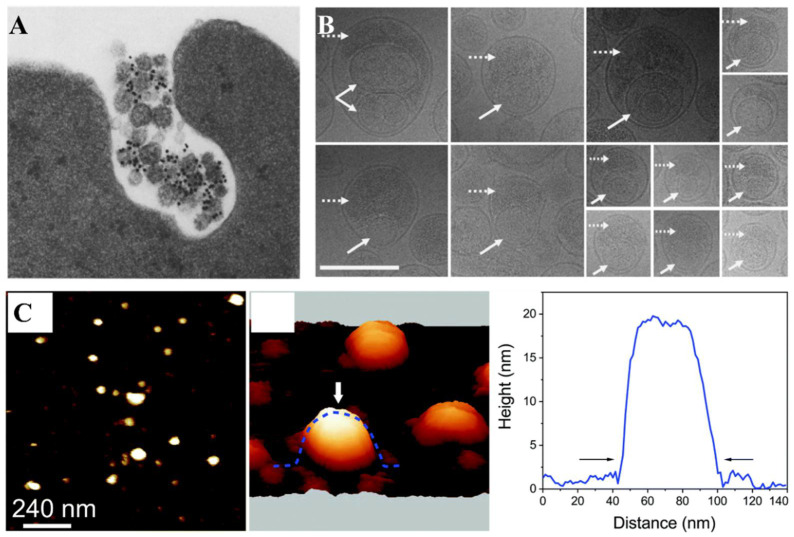
Electron microscopy-based methods for the single EV morphology characterization. (**A**) TEM image of small dense bodies labeled with gold nanoparticles released from multivesicular elements. Reprinted with permission from [40]. Copyright 1985, Rockefeller University Press. (**B**) Morphological characteristics of exosome-like vesicles observed by cryo-EM. Dashed white arrows are showing dark depositions, solid white arrows are showing secondary vesicles. Reprinted with permission from [42]. Copyright 2008, Wiley-Liss, Inc. (**C**) A 3D-force map of exosomes showing round-shaped objects. An exosome marked by a white arrow was used for the force mapping analysis. The exosome samples were isolated by ultracentrifugation. Reprinted with permission from [43]. Copyright 2018, Royal Society of Chemistry.

To reveal the morphology of the single EV, transmission electron microscopy (TEM) [35], cryo-EM [36] as well as atomic force microscopy (AFM) [37] have been widely used to observe EVs in different conditions. In fact, it was in the 1960s [38] that EVs were first-time observed under an EM when they were described as “platelet dust”. In the 1980s, Pan et al. [39,40] described EVs as small dense bodies observed under TEM with a size of 50 nm (Figure 1A) and demonstrated that the transferrin receptor was externalized in these vesicles. EVs exhibit a saucer-like structure under TEM caused by the collapse of the samples during sample drying treatment, while cryo-EM can completely preserve their original morphology, as they were imaged in their native aqueous status without fixation. In addition, cryo-EM enables the precise observation of the EV morphology and heterogeneity [41]. Poliakov et al. [42] reported the detailed structures of small exosome-like vesicles isolated from human seminal fluid for the first time (Figure 1B). They analyzed 301 cryo-EM images of vesicles purified by a sucrose gradient, and described their morphological characteristics in detail, including multiplicity, shape, external features and the overall density of the vesicles. 

In order to accurately analyze EVs in three dimensions, AFM was utilized to disclose the structural and nanomechanical features of EVs. Yurtsever et al. [43] found distinct local domains on the surface of exosomes using 3D-AFM (Figure 1C). The exosome samples were prepared by ultracentrifugation and a MagCapturerm exosome isolation kit. They revealed that exosomes have an elastic modulus ranging from 50 MPa to 350 MPa. Moreover, they also found that the exosome mechanical properties are related to the malignancy of tumor cells and confirmed that the increased elastic modulus of exosomes derived from metastatic tumor cells contained rich specific proteins related to the elastic fiber formation. These findings are important for future studies on exosome biofunctions and provide a different strategy for using exosomes as cancer diagnostic biomarkers. Ye et al. [44] employed AFM to investigate the physical properties of single EVs released by cancer cells. The EV samples were prepared by ultracentrifugation. The relationship between the tumor malignancy and the EV size was explored. The EVs of greater malignancy and smaller size exhibit an increased stiffness and osmotic pressure but a lower bending modulus, establishing a relationship between the tumor malignancy and EV nanomechanical signatures. 

### 2.2. Enumeration Techniques for Single EVs

**Figure 2:**
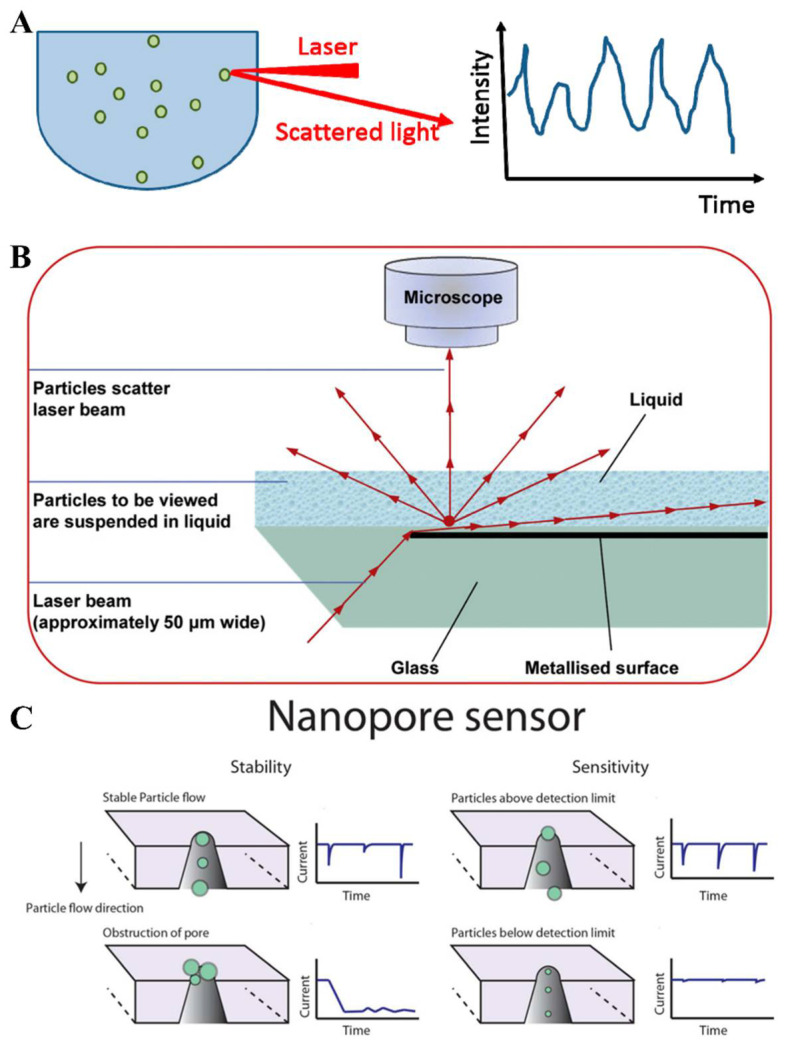
(**A**) DLS detects the scattered light intensity from the particles undergoing the Brownian motion in a solution that fluctuates over time. Reprinted with permission from [46]. Copyright 2017, Multidisciplinary Digital Publishing Institute. (**B**) Nanoparticle tracking analysis (NTA) utilizes the Brownian motion and light scattering to determine the particle size in solution. The scattered light from the particles is captured by a digital camera. Reprinted with permission from [49]. Copyright 2011, Elsevier. (**C**) In tunable resistive pulse sensing (TRPS), the pore size could be reversibly adjusted, which enables the flexible detection of single particles of different sizes. Reprinted with permission from [52]. Copyright 2015, American Chemical Society.

Since EVs are a group of nano- to micro-sized particles varying from 30 nm to 5000 nm, it is still difficult to accurately enumerate pure EVs. One of the most widely used techniques for the enumeration of mono-dispersed nanoparticles is dynamic light scattering (DLS) [45,46]. DLS detects the scattered light intensity from particles undergoing the Brownian motion in a solution that fluctuates over time (Figure 2A). The particle size is measured indirectly, based on the movement of the particles, so the resolution of the DLS [47] is limited when applied to characterize the polydisperse sample with heterogeneous EVs in size. A nanoparticle tracking analysis (NTA) [48,49] also utilizes the properties of the Brownian motion, as well as light scattering, to estimate the particle size in a solution. The scattered light from the particles is captured by a digital camera (Figure 2B), and then computer software is used to analyze the Brownian motion of each particle. This particle-by-particle analysis eliminates the inherent limitation in the DLS. Due to the unique advantage, the NTA has been favorably evaluated when used for the EV characterization.

Resistive pulse sensing (RPS) [50] is an efficient technique for the particle enumeration and size measurement in electrolyte solutions, based on the Coulter counter principle. A tiny insulating aperture is submerged in an electrolyte solution containing suspended particles. When a particle passes through the orifice, the changes, in terms of the ion current pulse, can be detected. The size of the particles that can be detected is determined by the diameter of the aperture. The use of RPS for single EV counting [51] attracts considerable interest. To improve the feasibility of the particle detection and the flexibility of the pore size, tunable resistive pulse sensing (TRPS) [52] was then proposed, and the pore size can be reversibly adjusted, which enables the flexible detection of single particles of different sizes (Figure 2C). Yet, the EV characterization using TRPS is still suffering from the heterogeneity problem and the unknown buffer components in the biological samples [53]. De Vrij et al. [54] spiked the internal control beads into EV samples for a variation correction, enabling the quantification of EVs in biological samples by a scanning ion occlusion sensing (SIOS) technology without EV labeling. This method provides a valuable strategy to the TRPS approach for the EV quantification.

**Figure 3:**
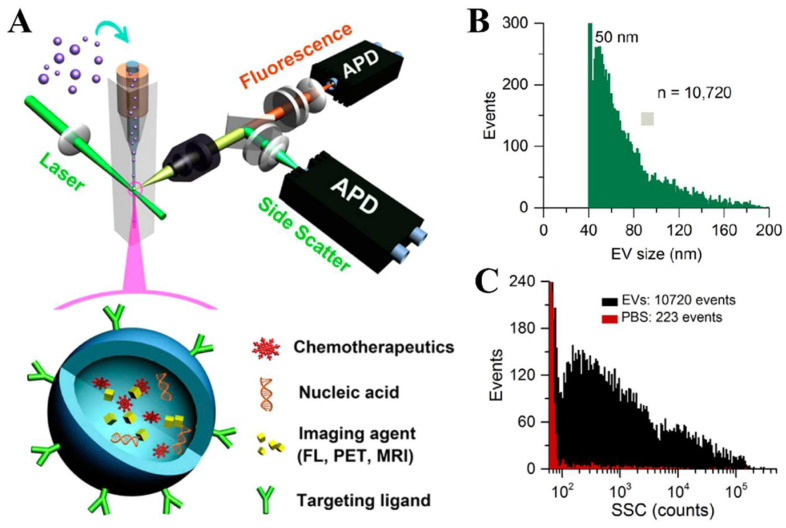
A high-resolution flow cytometry for the individual EV analysis. (**A**) Schematic diagram of a laboratory-built HSFCM system by Yan’s group. Reprinted with permission from [56]. Copyright 2014, American Chemical Society. (**B**) Histogram of the particle size for the EV sample by HSFCM (*n* = 10,720). Reprinted with permission from [57]. Copyright 2018, American Chemical Society. (**C**) Detection of the side scattering (SSC) of PBS (red) and EVs (black), collected by HSFCM over 2 min each. Reprinted with permission from [57]. Copyright 2018, American Chemical Society.

Despite the fact that the NTA and RPS have been requested for the EV characterization, they are still unable to accurately determine the EV size, due to the existence of complicated background nanoparticles in the EV samples, including lipoproteins and protein aggregates. Flow cytometry might be an ideal method for the EV analysis at the single particle level, to obtain more specific information. However, conventional flow cytometry was designed for the microparticle characterization, such as cells, which may be not suitable for the nano-sized EVs. Van der Vlist et al. [55] reported a protocol to set up a high-resolution flow cytometry for the individual nano-sized vesicle analysis. In this method, the cell-derived vesicles are labeled with bright fluorescent, then an optimized configuration of the flow cytometer was employed for the flow cytometric analysis. The high-resolution flow cytometer enables the precise identification and analysis for the nano-sized vesicles, as small as 100 nm in diameter. Yan’s group [56,57] built a high-sensitivity flow cytometer (HSFCM) (Figure 3A) that achieved a limit of detection for single gold nanoparticles of 7 nm. They then employed the HSFCM for the single EV detection that can detect EVs between 40 nm and 200 nm in diameter with an analysis rate of 10,000 particles/min (Figure 3B,C). HSFCM offers a sensitive way to measure the size of single EVs and analyze the surface proteins, which might considerably enhance the understanding of the cell-cell communication mediated by EVs and facilitate the development of new diagnostic techniques. 

**Figure 4:**
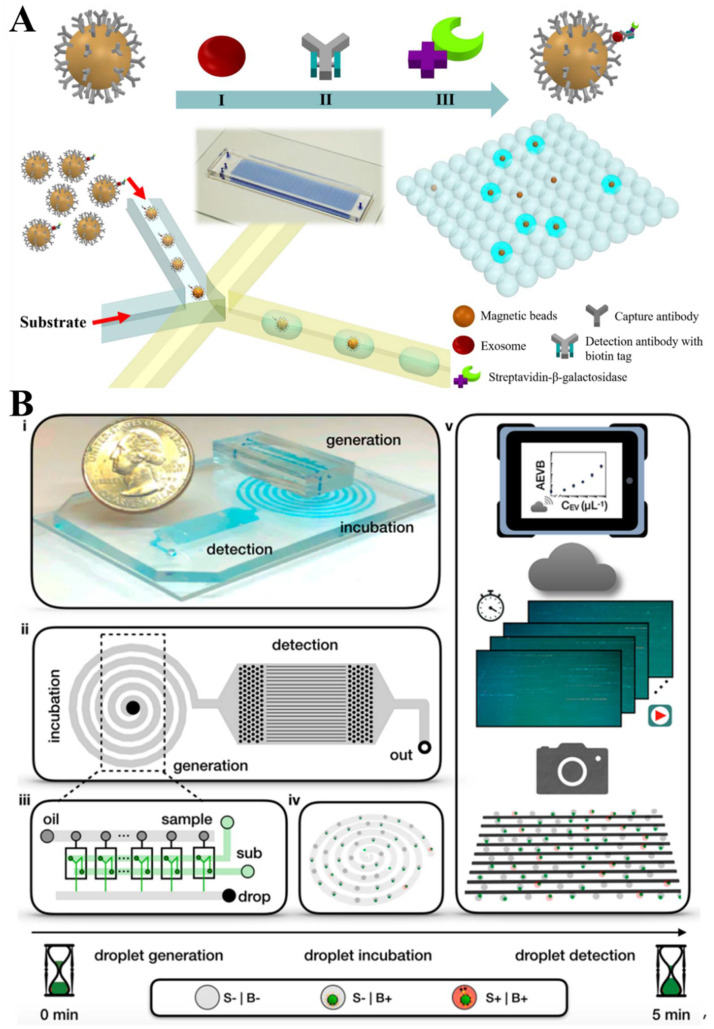
(**A**) The droplet-based single-exosome-counting enzyme-linked immunoassay (droplet digital ExoELISA) approach for the single EV counting. Reprinted with permission from [59]. Copyright 2018, American Chemical Society. (**B**) Single EV detection on a droplet-based extracellular vesicle analysis (DEVA) assay. Reprinted with permission from [60]. Copyright 2022, American Chemical Society.

Lately, droplet technology [58] has been used for the counting of single EVs, showing a dramatic improvement in the detection sensitivity. Liu et al. [59] reported an immunosorbent test for the digital validation of the target EVs (Figure 4A). Briefly, EVs were first captured on the magnetic beads and labeled with a reporter which generates a fluorescent signal. These beads were then encapsulated separately into droplets with just one bead enclosed in each droplet. The droplet-based single-exosome-counting enzyme-linked immunoassay (droplet digital ExoELISA) achieved a limit of detection, down to 10 exosomes/μL in an absolute counting way for the single exosomes. Compared with the signal amplification method of the ELISA, the signal amplification method of DNA in an in vitro amplification, may be more stable. They successfully quantified EVs in serum samples using a droplet digital ExoELISA. Yang et al. [60] developed a droplet-based extracellular vesicle analysis (DEVA) assay (Figure 4B), which enables the quantification of EV subpopulations with a high throughput of 20 million droplets per minutes, which is 100 times greater than microfluidic systems. Notably, a LOD of 9 EVs/μL was achieved when processing EVs in the PBS samples. The application of the droplet digital technology provides a completely new way that allows for the ultrasensitive EV detection.

### 2.3. Techniques for the Single EV Molecular Analysis

The techniques for the molecular analysis of bulk EVs have been widely reported, such as the western blot (WB) and the enzyme linked immunosorbent assay (ELISA), but it is limited in the analysis of the surface proteins or nucleic acids for the single EVs, due to the low detection sensitivity and the heterogeneity of the single EVs. Here, we summarize the advances of the current technologies developed for the molecular analysis at the single EV level.

#### 2.3.1. High-Sensitivity Flow Cytometer

**Figure 5:**
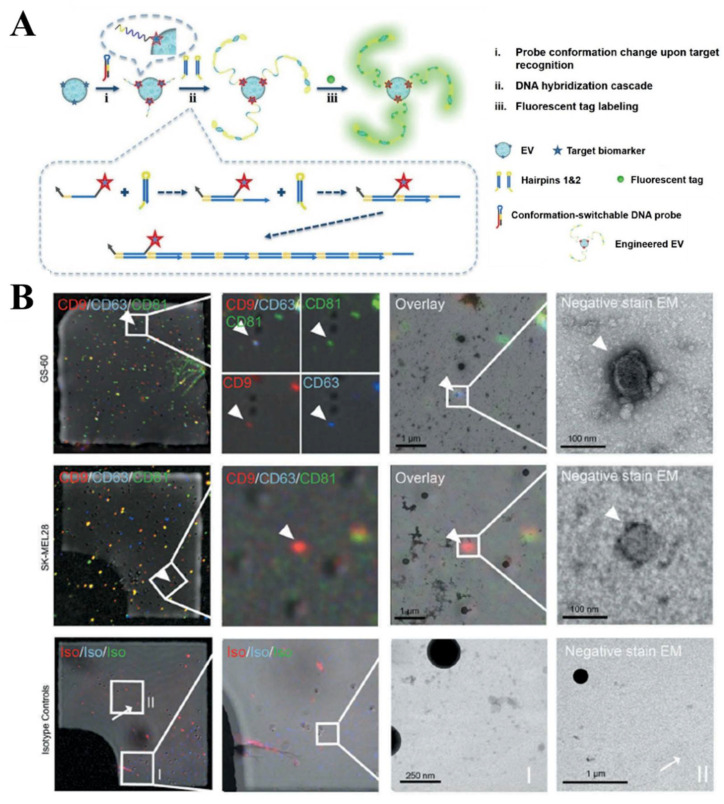
Flow cytometry-based techniques for the single EV molecular analysis. (**A**) Single EV analysis using FCA, achieved by the target-initiated engineering of the DNA nanostructures. Reprinted with permission from [32]. Copyright 2018, Wiley-VCH Verlag GmbH & Co. KGaA, Weinheim. (**B**) Representative IFCM for the tetraspanin profiles on different EVs. White arrowheads indicate fluorescence positive single EVs and mark an example of a triple positive EV (GS-60) as well as an example of a single positive EV (SK-MEL28). White arrows indicate fluorescence positive events that do not appear as EV-like structures in TEM. Reprinted with permission from [61]. Copyright 2019, Taylor & Francis Group.

Although there is an intrinsic limitation on the detectable size for flow cytometry, when directly detecting exosomes, it has been extensively used in studying the surface marker on exosomes through various instrumentation development, as well as exosome labeling strategies. Shen et al. [32] designed a probe with switchable conformations that recognizes CD63, to visualize individual EVs (Figure 5A). The anti-CD63 aptamer was introduced in the probe for the target recognition, and a trigger domain was designed for initiating the DNA growth via the hybridization chain reaction (HCR). Thereby, the DNA nanostructures resulting from the target-initiated engineering (TIE) could enlarge the labeled EVs, making them detectable by a confocal fluorescence microscope. A simultaneous analysis of the dual surface marker expression on a single EV was also demonstrated in this study. The multiparameter analysis for the single EVs could also been achieved by using imaging flow cytometry (IFCM) (Figure 5B) [61]. IFCM facilitates the analysis of the surface protein profiles of CD9, CD63 and CD81 on glioblastoma EVs. They found that the number and proportion of CD63+ and CD81+ EVs in cancer patients, were significantly increased. 

#### 2.3.2. Raman Spectroscopy-Based Technique

**Figure 6:**
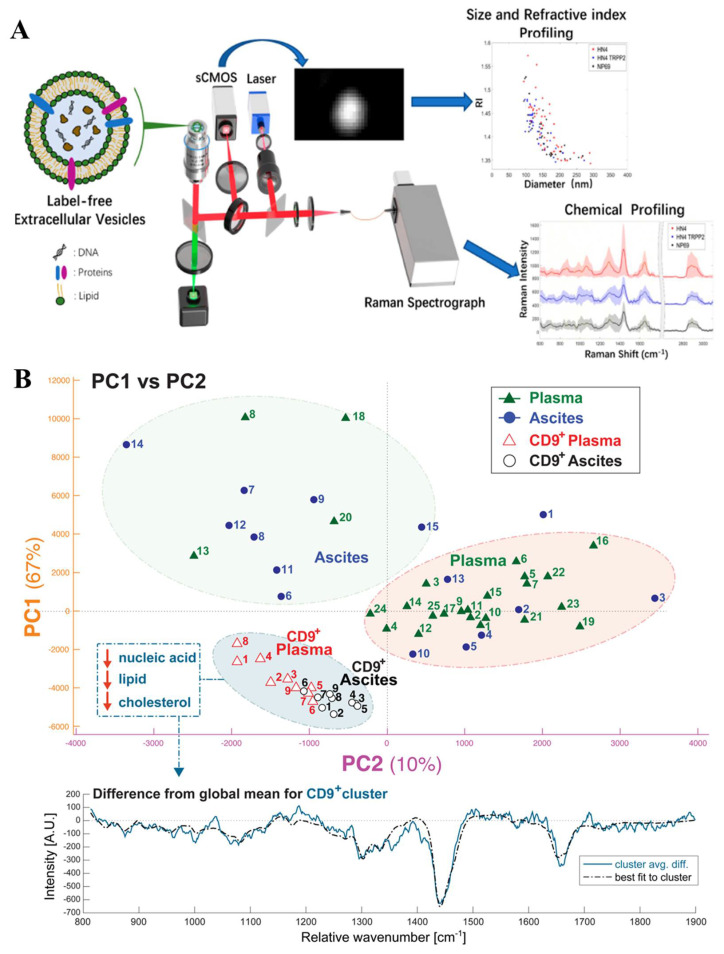
Raman-based methods for the single EV molecular analysis. (**A**) Raman−enabled nanoparticle trapping analysis (R−NTA) for the EV analysis, by using morphology and chemical information. Reprinted with permission from [65]. Copyright 2020, American Chemical Society. (**B**) A multispectral optical tweezers (MS−Ots) for the single vesicle molecular fingerprinting. Reprinted with permission from [66]. Copyright 2017, American Chemical Society.

Surface-enhanced Raman scattering (SERS) [62] is a potent surface-sensitive approach that can analyze the molecular spectral signals, even at the level of a single molecule, by a multi-orders-of-magnitude amplification. Tirinato et al. [63] used SERS to analyze the healthy cell-derived EVs and tumor cell-derived EVs, with a super-hydrophobic nanostructured substrate, that can control diluted solutions precisely. Tumor cell-derived exosomes, exhibiting a richer RNA content, can be differentiated from healthy cell-derived exosomes specifically. Raman tweezers microspectroscopy (RTM), which combines optical trapping with Raman probing, provides genuine Raman fingerprints of the sample’s constituent biomolecules. Kruglik et al. [64] demonstrated the utility of RTM in defining different subpopulations of exosomes. Notably, RTM provides a universal molecular signature for different EV subpopulations at the single-EV level. A Raman-enabled nanoparticle trapping analysis (R-NTA), proposed by Dai et al. [65], presented another method to access the chemical composition at the level of a single particle in a label-free way (Figure 6A). They demonstrated the power of the R-NTA platform to characterize the morphology, as well as the chemical heterogeneity of the nanoparticles. Carney et al. [66] described the first application of the multispectral optical tweezers (MS-OTs) for the individual vesicle molecular profiling. This platform has the unique capability to multiplexing quantify the compositional difference across the EV groups (Figure 6B). 

#### 2.3.3. Single Particle Interferometric Imaging Sensing (SP-IRIS) Technology

**Figure 7:**
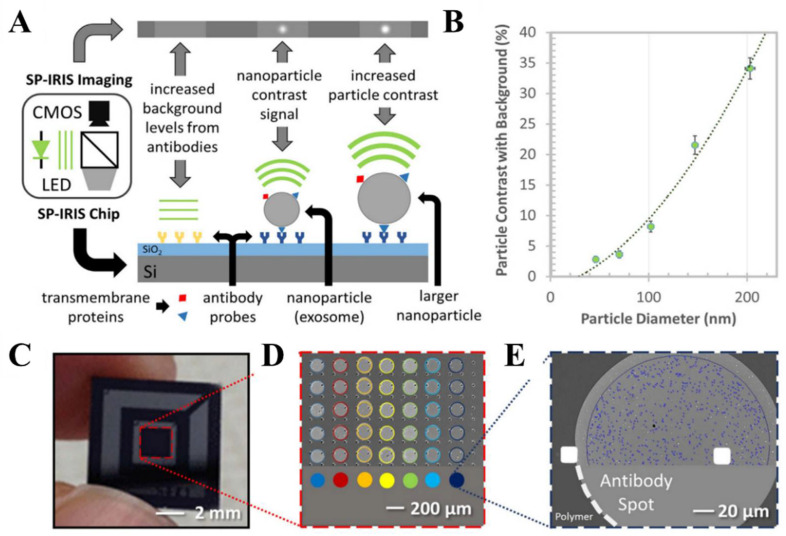
Schematic representation of the single particle interferometric imaging sensing (SP-IRIS) detection process. (**A**) Detection principle of SP-IRIS technique. (**B**) SP-IRIS signal for polystyrene nanoparticles (50–200 nm). (**C**) Image of a SP-IRIS chip. (**D**) Microarray of immobilized capture probes. (**E**) SP-IRIS image of a capture probe. Reprinted with permission from [76]. Copyright 2016, Springer Nature.

Single particle interferometric imaging sensing (SP-IRIS) [67,68,69,70,71] is of performance with the single-molecule detection capability. It enables the nanoparticle size measurement of 70 nm from complex biological samples, at low concentrations [72,73]. Recently, the potential of SP-IRIS on the single EV characterization, has been explored [74,75]. EVs are first isolated using immune recognition, and then the surface proteins and the RNA/DNA contents of the target EV are quantified. The technique has been shown to fractionate subpopulations of exosomes with specific markers, allowing for a better understanding on their heterogeneity (Figure 7) [76]. An et al. [77] performed a preliminary screening of the common EV biomarkers CD9, CD63 and CD81 tetramers, using SP-IRIS. It was found that the CD81 expression levels were significantly higher in all EV samples, compared to CD9 and CD63. 

#### 2.3.4. Atomic Force Microscope—Infrared Spectroscopy (AFM-IR) 

**Figure 8:**
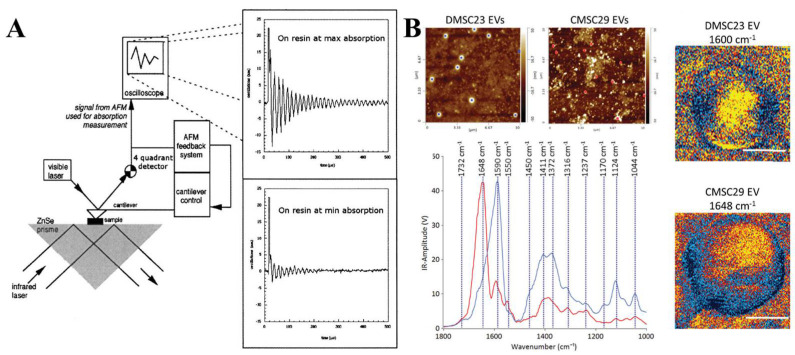
(**A**) Experimental setup of the first AFM−IR spectroscopy. Reprinted with permission from [82]. Copyright 2005, Optical Society of America. (**B**) The height images and averaged spectra generated from AFM-IR for two different EV populations. Averaged AFM−IR spectra of DMSC23 EVs (blue) and CMSC29 EVs (red). Reprinted with permission from [89]. Copyright 2018, Royal Society of Chemistry.

Taking advantage of the high-resolution of AFM, individual EVs can be probed for the identification of their nanoscale composition [78,79,80]. The quantitative differences analyzed at the single-vesicle level between the normal and patient’s exosomes have been reported using high-resolution AFM [81]. Dazzi et al. [82,83,84] developed a technique coupling AFM and IR spectroscopy (AFM-IR) (Figure 8A), which enabled thenanoscale chemical component analysis [85,86]. AFM-IR offers the tremendous potential to detect the biomolecules inside individual EVs without labeling. The height images from AFM and AFM-IR spectra allow for the direct comparison of two different EV populations (Figure 8B), which can be accomplished on a single EV [87,88,89]. In addition, the IR maps for the different EVs enable AFM-IR to generate specific fingerprints for individual EVs. AFM-IR is currently the most sensitive technique to investigate the heterogeneity across individual EVs and also EV subpopulations. 

#### 2.3.5. Droplet Digital Polymerase Chain Reaction (ddPCR) Technology

**Figure 9:**
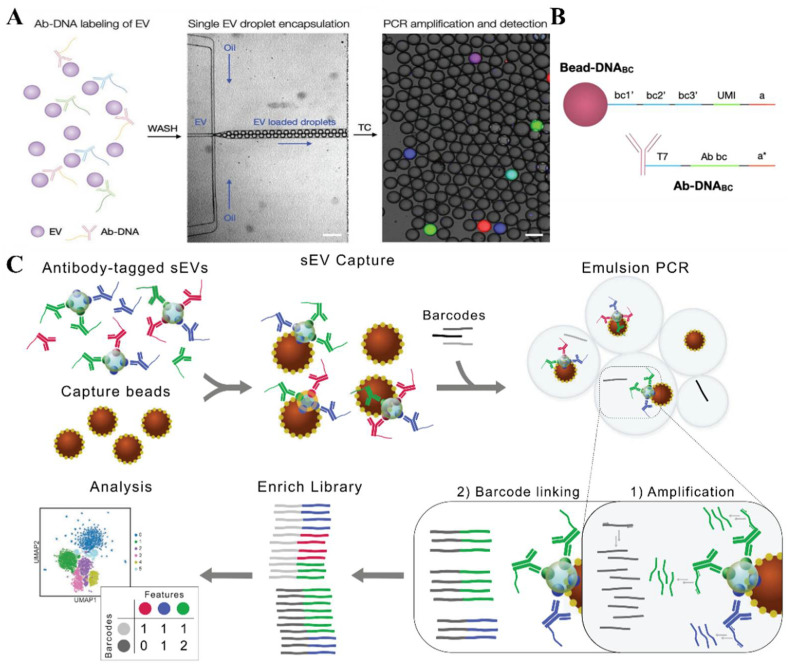
Droplet digital technique for the single EV molecular analysis. (**A**) Schematic of the droplet-based single EV detection. Labeled EVs are detected in the form of fluorescent droplets. Reprinted with permission from [90]. Copyright 2020, WILEY-VCH Verlag GmbH & Co. KGaA, Weinheim. (**B**) DNA sequence composition on the barcoded beads and antibodies used for seiSEQ. Reprinted with permission from [91]. Copyright 2021, American Chemical Society. (**C**) Droplet barcode sequencing principle for the multiplexing protein analysis assays for the quantification of the surface proteins on individual vesicles. Reprinted with permission from [93]. Copyright 2022, Taylor & Francis Group.

The Weissleder group [90,91,92] exploited the application of the antibody-based immuno-droplet digital polymerase chain reaction (iddPCR) for the protein analysis on single EVs. Followed by the PCR amplification, individual droplets with target EVs in them were imaged by fluorescence microscopy for the EV quantification (Figure 9A). To specify the limitation from the fluorescence readout, they proposed an antibody-DNA barcode-based immunosequencing method (seiSEQ) [91]. The EVs attached with Ab-DNA are subsequently encapsulated into 70 µm droplets along with barcoded beads (Figure 9B), where the PCR amplifies the DNA barcode signals, enabling the multiplexing of the protein profile in individual EVs with a high specificity. Banijamali et al. [93] further developed a droplet barcode sequencing technology to analyze multiple proteins without barcoded gel beads (Figure 9C). The droplet digital technology has also been employed to amplify and characterize single EV nucleic acids. The traditional method for the EV nucleic acid analysis requires the EV lysis, followed by the quantitative real-time PCR (qPCR) or ddPCR in a population-based analysis. Pasini et al. [94] have reported that the droplet-based method enabled the detection of nucleic acid biomarkers in a single EV, which may explore more information on the specific EV subset that contains specific biomarkers.

Additionally, there are several further innovative strategies that have been proposed for the EV molecular analysis. Huang et al. [95] demonstrated a single EV counting system using lanthanide-doped upconversion nanoparticles (UCNPs). They showed the significant potential of the UCNPs to “digitally” quantify the surface proteins on the individual EVs, which provides an approach to monitor the EV heterogeneity changes during the tumor’s progression [96]. Dittrich’s team [97] fabricated a microfluidic platform that can capture, quantify and classify the EVs released by a single cell. Each detected EV can be assigned to one of 15 unique populations through multicolor immunostaining. This study revealed the presence of highly heterogeneous phenotypes of EVs from one single cell. 

In summary, we listed the comparison of the different analysis techniques for single particles, described below in Table 1. 

## 3. Clinical Applications of the Single EV Analysis in Early Cancer Detection

EVs have been considered as a tumor marker produced by tumor cells or other cells, in response to tumors throughout the development, occurrence and proliferation of malignant tumors, reflecting the existence and progression of tumors. It is crucial to detect the very limited signal in the early stages of cancer amid the noise of normal human biology. The single EV analysis provides a promising method for developing tumor markers, due to its unique sensitivity and specificity. In this section, we highlight several applications of the single EV analysis, including the subpopulation analysis, protein profiling and genetic analysis. An overall summary is shown in Table 2.

### 3.1. Subpopulation Analysis

**Figure 10:**
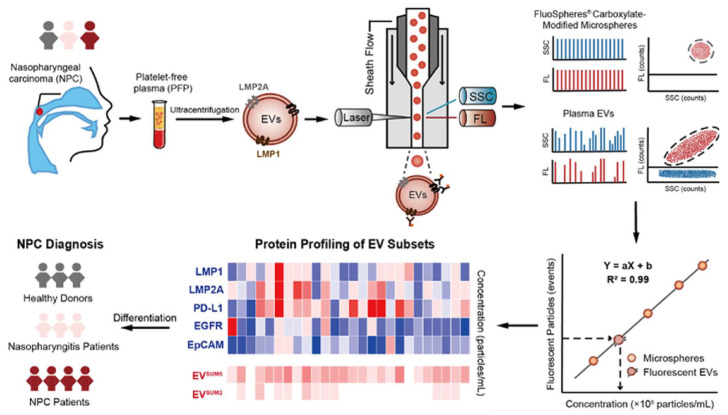
Phenotypic analysis of the plasma EVs for the detection of nasopharyngeal carcinoma. The exosome samples were isolated from plasma by ultracentrifugation. Reprinted with permission from [100]. Copyright 2022, American Chemical Society.

The single EV analysis provides a powerful tool to explore the diversity and heterogeneity of EVs that help to understand their precise role in the development of tumors, which will ultimately promote the analysis of EVs as a diagnostic marker. Hu et al. [100] quantified the concentrations of five EV subsets by examining the expression of the surface proteins (LMP1, LMP2A, PD-L1, EGFR and EpCAM) on individual EVs in plasma samples, using nanoflow cytometry (nFCM)-based single particle enumeration (Figure 10). The clinical utility of LMP1(+) and LMP2A(+) EVs was investigated for the diagnostic capacity of the NPC and NPG patients, and an accuracy of 82.6% was achieved. They then developed a five-marker sum signature that could discriminate the NPC patients from both healthy people (accuracy = 96.3%) and NPG patients (accuracy = 83.1%), respectively, significantly surpassing the traditional VCA-IgA assay.

**Figure 11:**
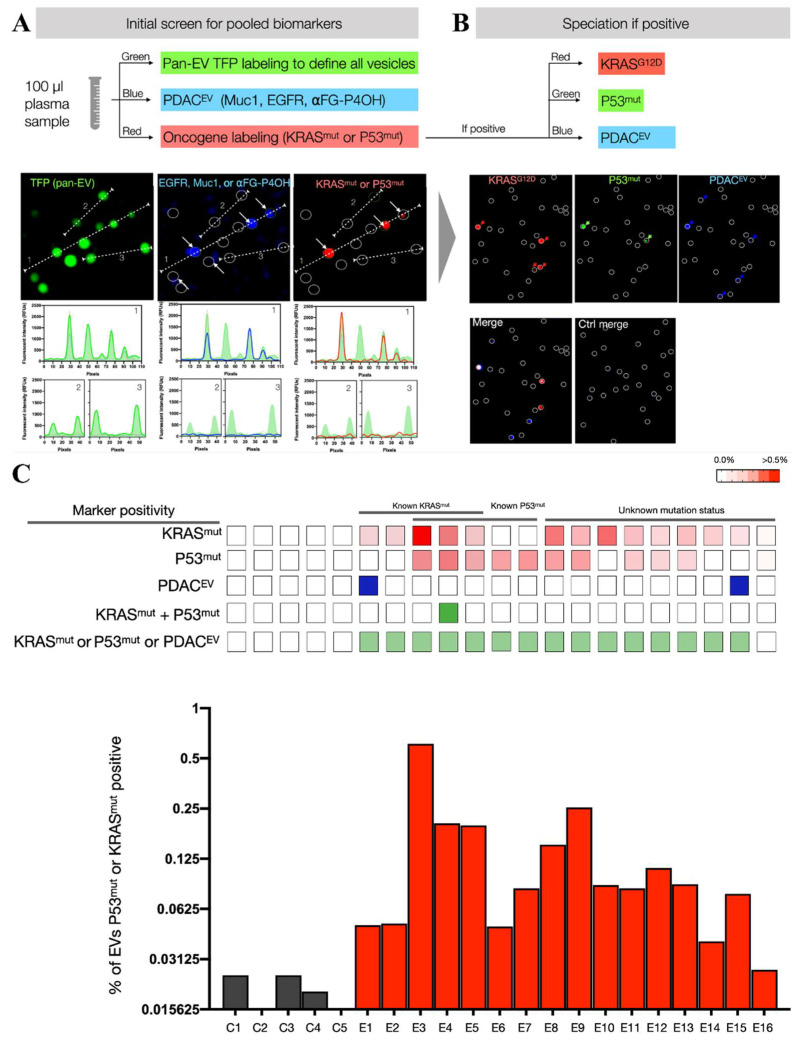
Workflow of the single EV analysis. (**A**) Target EVs are labeled for the pooled biomarkers. (**B**) A separate fluorochrome defined EV in each positive case. (**C**) Analysis of KRAS^mut^ and P53^mut^ in EVs from clinical samples. Reprinted with permission from [103]. Copyright 2022, American Association for the Advancement of Science.

In Weissleder’s recent study [103], they performed multiplexed protein measurements in individual EVs and a composition analysis of the putative cancer markers in pancreatic cancer (Figure 11). In a blinded study, KRAS^mut^ and P53^mut^ proteins were detectable in 15 of 16 patients with stage I PDAC by the single EV analysis (sEVA) approach, which is typically in <0.1% of vesicles. They estimated that the sEVA approach had a detection limit of 0.1 cm^3^ tumor volume for the PDAC patients, which is below the capability of clinical imaging. Compared to the bulk-based EV analysis, the single EV analysis could exclude the contamination from plasma proteins, ensuring a more specific and accurate detection. The development of a single EV-based quantitative analysis platform provides a more sensitive tool to explore the application potential in cancer detection.

### 3.2. Protein Profiling

**Figure 12:**
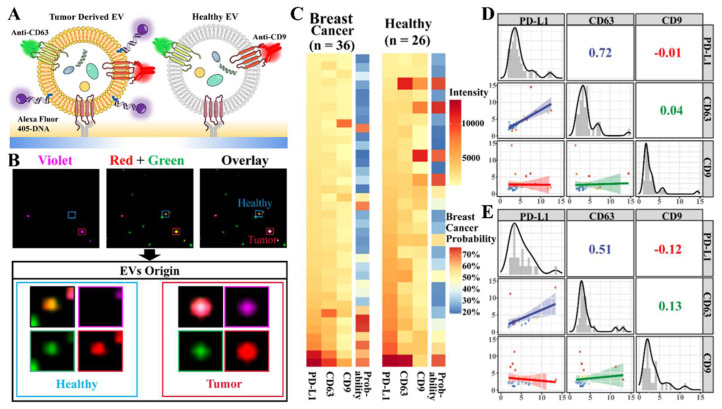
Intelligent probabilistic system for the single EV analysis facilitating the tracing cellular origin. (**A**) Schematic illustration for the analysis of healthy and tumor EVs, using the single EV analysis. (**B**) TIRFM images of the mixed EV samples. (**C**) Phenotyping of the EVs in clinical plasma samples. (**D**,**E**) Correlation analysis of the PD−L1, CD63 and CD9 expressions for EVs from clinical cancer plasma samples (**D**) and healthy plasma samples (**E**). Reprinted with permission from [106]. Copyright 2021, American Chemical Society.

EV proteins could reflect the status of a tumor, providing important information on the tumor biology. Xiao et al. [106] analyzed the tetraspanin expression profiles on EVs that were dual stained by fluorescent antibodies, and identified their cellular origin. They found a substantial association between the CD63 and PD-L1 expression in the EVs from breast cancer patients with a Pearson correlation coefficient of 0.72 (Figure 12). Chen et al. [108] developed a DNA points accumulation for imaging in the nanoscale topography (DNA-PAINT) based quantitative platform, and profiled four surface biomarkers on single exosomes (HER2, GPC-1, EpCAM and EGFR). The exosomes were first isolated from the serum samples by a precipitation method. These DNA-PAINT methods could detect pancreatic cancer and breast cancer blindly with an accuracy of 100%. Multiple protein profiling for single EVs provided a more promising diagnostic potency in early cancer detection.

### 3.3. RNA Analysis

**Figure 13:**
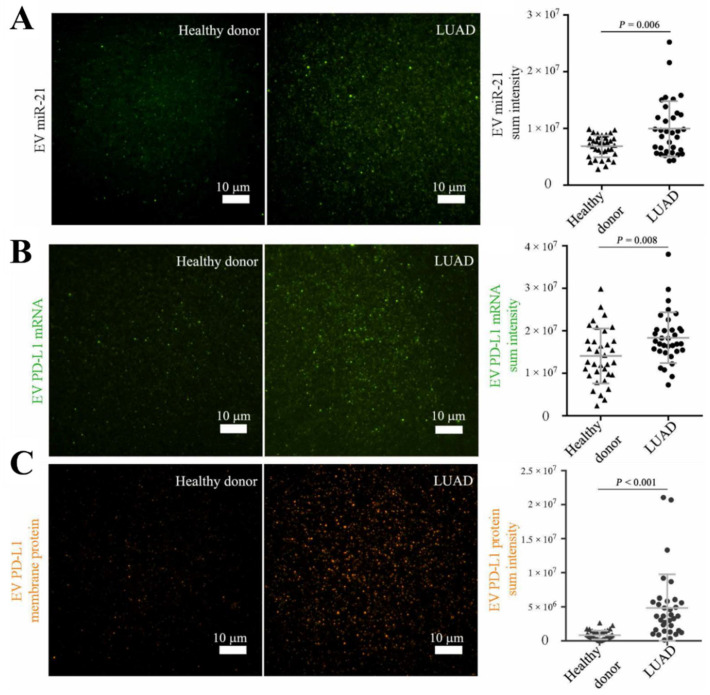
Measurement of the different EV cargo biomarker expressions. Representative images and analysis of the (**A**) EV miR-21, (**B**) EV PD-L1 mRNA and (**C**) EV PD-L1 membrane protein. Reprinted with permission from [110]. Copyright 2020, American Association for the Advancement of Science.

The benefit from the protection of the EV membranes, the reserved RNA inside them is stable and contains rather intact information, compared to the circulating RNA. Studies on the tumor EV-derived RNA may reveal the molecular mechanism of the specific RNAs in tumor development. Zhou et al. [110] developed a Nano-bio Chip Integrated System for Liquid Biopsy (HNCIB) system to detect proteins and RNA in EVs, simultaneously, at the level of the single vesicles. This HNCIB system could differentiate the patients with lung adenocarcinoma (LUAD) from healthy donors by detecting the up-regulated expression of the EVs’ cargo biomarkers (Figure 13). They then used machine learning methods to improve the detection accuracy in the image processing.

## 4. Conclusions and Outlook

Although EVs have been considered as one of the most promising biomarkers that carry rich diagnostic information, there are still two critical challenges in EV-based cancer detection: (i) the ultra-low amount and purity in early stage and (ii) high heterogeneity. Consequently, the development of more accurate methods for the EV characterization is highly in demand. We reviewed the current development of the single EV technologies for the EV physical characterization, EV counting and EV molecular profiling, in light of clinical requirements. Microscopy-based methods, including TEM, cryo-EM and AFM, are commonly employed for EV study. New strategies, such as nFCM and droplet techniques, enabled the specific EV enumeration at a single particle level, which would dramatically increase the sensitivity of the tumor-derived EV detection. In order to reveal the heterogeneity of EVs, more efforts are focused on the improvement on the molecular analysis for the individual EVs. The feasibility of the protein profiling and RNA/DNA testing has been demonstrated by several techniques. Especially, the advances in the multiplexing protein profile on the single EV pave the way to the high throughput analysis of the tumor EVs with a high sensitivity and specificity. Despite the advances, translating them into clinical application remains difficult. The reliability is a serious issue, because of the lack of normalization on the clinical sample pretreatment and the data analyses. Repeatability and more clinical validation would be necessary for the future development of emerging technologies for the single EV analysis.

## Figures and Tables

**Table 1:** Comparison of the different vesicle analysis techniques.

Technology	Advantages	Disadvantages	Time Required per Measurement	Limit of Detection	Ref.
DLS	Rapid; Samples are reusable	Not suitable for polydispersed particles; High sample purity required	10 min	10^5^ EVs μL^−1^	[47]
NTA	Rapid; Samples are reusable	High sample purity required	10 min	10^5^ EVs μL^−1^	[49]
TRPS	Suitable for polydispersed Samples; Single particle detection	Influenced by membrane pore size, shape and vesicle surface property; Membrane clogging	Few minutes	10^2^ EVs μL^−1^	[52]
nFCM	Single particle detection; Rapid	High cost	Few minutes	10^4^ EVs μL^−1^	[57]
Digital droplet Technology	Single particle detection; Low detection limit	Time-consuming	30 min	10 EVs μL^−1^	[59]

**Table 2:** Clinical applications of the single EV analysis in early cancer detection.

EV Analysis	Target Type	Multiplexing	Biomarkers	Cancer Type	Sources	Patient Number	Detection Methods	Diagnostic Performance	Year	Ref.
subpopulation	protein	no	CD147 (+) EVs	Colorectal cancer	Plasma	N = 37	nFCM	CRC vs. HD, ROCAUC = 0.932	2018	[57]
subpopulation	protein	no	GPC-1 (+) exosomes	Breast cancer	Serum	N = 12	droplet digital ExoELISA	N/D	2018	[59]
subpopulation	protein	yes	CD63/EpCAM/MUC1-triple-positive EVs	Breast cancer	Plasma	N = 14	surface plasmon resonance (SPR)	BrCa vs. HD, accuracy = 91%	2020	[98]
subpopulation	protein	yes	CD9-CD63 (+) EVs, PD-L1-CD63 (+) EVs	Large B-cell lymphoma	Plasma	N = 164	single molecule array technology (SiMoa)	LBCL vs. HD ROCAUC = 0.99	2021	[22]
subpopulation	protein	yes	CD63 (+) EVs, THBS2 (+) EVs, VCAN (+) EVs, TNC (+) EVs	Lung cancer	Plasma	N = 22	SERS	ROCAUC = 0.85	2022	[99]
subpopulation	protein	yes	5 EV subsets of LMP1, LMP2A, PD-L1, EGFR, EpCAM	Nasopharyngeal cancer	Plasma	N = 42	nFCM	NPC vs. HD, accuracy = 96.3%. NPC vs. NPG, accuracy = 83.1%.	2022	[100]
subpopulation	protein	no	EGFR (+) EVs, CA19-9 (+) EVs	Pancreatic cancer	Plasma	N = 5	quantitative single molecule localization microscopy (qSMLM)	N/D	2019	[101]
subpopulation	protein	no	LRG-1 (+) EVs, GPC-1 (+) EVs	Pancreatic cancer	Serum	N = 15	SERS	ROCAUC= 0.95, sensitivity = 90.0%, specificity = 86.7%	2022	[102]
subpopulation	protein	yes	KRAS^mut^ and/or P53^mut^ positive EVs	Pancreatic cancer	Plasma	N = 16	high-resolution microscopy	early stage PDAC, accuracy = 15/16	2022	[103]
protein	protein	no	CD47	Breast cancer	Serum	N = 60	micro flow cytometry (MFC)	N/D	2016	[104]
protein	protein	no	MUC5AC	Intraductal papillary mucinous neoplasms	Plasma	N = 133	FCM	Sensitivity = 82%, specificity = 100%	2020	[105]
protein	protein	yes	PD-L1, CD9, CD63	Breast cancer	Plasma	N = 36	total internal reflection fluorescence (TIRF)	N/D	2021	[106]
protein	protein	yes	CD9-CD63, EpCAM-CD63	Colorectal cancer	Plasma	N = 163	single molecule array technology (SiMoa)	CD9-CD63, AUC = 0.96; EpCAM-CD63, AUC = 0.90;	2020	[107]
protein	protein	yes	HER2, GPC-1, EpCAM, EGFR	Pancreatic cancer, Breast cancer	Serum	N = 7, N = 7	DNA points accumulation for imaging in nanoscale topography (DNA-PAINT)	PaCa, accuracy =100% BrCa, accuracy = 100%	2019	[108]
RNA	RNA	no	PSA mRNA	Prostate cancer	Serum	N = 42	DNA tetrahedron-based thermophoretic assay (DTTA)	PCa vs. benign prostatic hyperplasia ROCAUC = 0.93	2021	[109]
RNA, protein	RNA, protein	yes	miR-21, PD-L1	Lung cancer	Plasma	N = 34	high-throughput Nano-bio Chip	N/D	2020	[110]
RNA, protein	RNA, protein	yes	PD-1, PD-L1, PD-1 mRNA, PD-L1 mRNA	Lung cancer	Serum	N = 54	total internal reflection fluorescence (TIRF)	Accuracy = 93.2%	2022	[111]

## Data Availability

Not applicable.
